# Bio-control efficacy of selected indigenous nematophagous fungi against *Meloidogyne enterolobii* in vitro and on dry bean (*Phaseolus vulgaris* L.)

**DOI:** 10.1007/s10123-024-00571-1

**Published:** 2024-08-10

**Authors:** Ndivhuwo Ramatsitsi, Zakheleni Palane Dube, Khosi Ramachela, Tuelo Motloba

**Affiliations:** 1 School of Agricultural Sciences, North-West University, Private Bag X2046, Mmabatho, 2745 South Africa; 2 School of Biology and Environmental Sciences, University of Mpumalanga, Private Bag X11283, Mbombela, South Africa; 3 Food Security and Safety Niche Area, Crop Science Department, North-West University, Private Bag X2046, Mmabatho, 2745 South Africa; 4 Forestry and Agricultural Biotechnology Institute, University of Pretoria, Private Bag X20, Hatfield, 0028 South Africa

**Keywords:** Compost, Parasitism, Reproductive factor, Root-knot nematodes

## Abstract

Dry bean (*Phaseolus vulgaris* L.) is an important commercialized field crop in South Africa for aiding in food security as a cheap protein source. However, it is highly susceptible to root-knot nematodes (RKN), *Meloidogyne* species. Use of indigenous nematophagous fungi as bio-control agents (BCA) of *Meloidogyne* nematodes is a promising research focus area. This is because indigenous fungal species are naturally part of the ecosystem and therefore compatible with other biological processes unlike most synthetic chemicals. The objective of the study was to identify indigenous nematophagous fungal BCA and establish their potential efficacy in reducing *M. enterolobii* population densities on dry bean with and without incorporation of compost. Screened indigenous fungal species included *Aspergillus terreus*, *Talaromyces minioluteus*,* T*. *sayulitensis*, *Trichoderma ghanense*, and* T*. *viride.* There were observed significant parasitism differences (*P* ≤ 0.05) among the BCA, with *T*. *ghanense* showing the highest egg parasitism (86%), followed by *T*. *minioluteus* (72%) and *T*. *sayulitensis* (70%). On the other hand, the highest J2 parasitism was observed on *T*. *minioluteus* (95%), followed by *A. terreus* and *T. viride* (63%). A similar trend was observed under in vivo conditions, with higher efficacy with compost incorporation. This provides a highly encouraging alternative and ecologically complementary *Meloidogyne* management in dry bean production.

## Introduction

Plant-parasitic nematodes have been a constraint to crop production since the early ages of cultivation (Moens et al. 2009). *Meloidogyne* species, root-knot nematodes (RKN), are particularly the most devastating as reviewed by Coyne et al. (2018). This could be credited to the species’ mode of feeding, wide host range (Agenbag 2016; Coyne et al. 2018), reproductive fecundity and short life cycles (Singh et al. 2013). Worldwide annual yield loss of 12.3%, approximately US$157 billion, is attributed to this genus alone (Singh et al. 2015). *Meloidogyne enterolobii*, a highly virulent and aggressive invasive species, is becoming increasingly economically significant around the globe (Philbrick et al. 2020). This species shows similar devastation as its counterparts, *M. incognita* and *M. javanica*, and has been noted to have the ability to overcome host resistance (Brito et al. 2020; Kiewnick et al. 2009) making it stand out with potentially severe crop damage.

Dry bean (*Phaseolus vulgaris* L.) is one of the important vegetable crops in South Africa (SA), majorly produced in the following Provinces: Free State (16600t), Limpopo (16000t), Mpumalanga (9700t) and North West (9300t) (DALRRD 2022). As of the 2020/21 production season, total area planted was 47 000 ha, from which R888 million was realized (DALRRD 2022) ranking it a second legume after soybean. The crop is grown for its dry seeds which can have up to 25% protein (Sathe 2002) exceeding that found in other crops such as maize, wheat, and rice (Shewry 2007), making it an affordable protein source. This legume is further used in animal feed formulations (Mahmoud et al. 2020; Osmane et al. 2017) and soil improvement purposes (DAFF 2010; Kebede 2021; Stagnari et al. 2017) through its endosymbiotic relationship with *Rhizobium* species that fix nitrogen. Most commercialized dry bean cvs. “Tygerberg,” “Kranskop,” ‘Kranskop-HRI,” “RS6,” and “DBS-360″ in SA are susceptible to *Meloidogyne* species (Pofu et al. 2019)*.*

Existing management measures such as the use of synthetic chemical nematicides are gradually being phased out due to their negative synergistic effects on the environment (Chen et al. 2020). Currently, there is growing interest in the use of bio-control agents (BCA) for management of *Meloidogyne* species (Ahmed et al. 2023; Baazeem et al. 2022; Patil et al. 2021). There is reported success on managing *Meloidogyne* species with fungal BCA on several commercial crops including tomato (*Solanum lycopersicum* L.) (Silva et al. 2017), brinjal (*S*. *melongena* L.) (Baazeem et al. 2022), cucumber (*Cucumis sativus* L.) (Naz et al. 2021), soybean (*Glycine max* L.) (Messa et al. 2020), and many others as reviewed by Peiris et al. (2020). Thus, indicating the potential of managing *M. enterolobii* on dry bean with indigenous fungal BCA. These eukaryotes have a selective adaptation to different soil and climatic conditions such as different temperature, pH and salinity levels (Pang et al. 2020; Ramatsitsi et al. 2023). Taking into consideration all these factors, it can be inferred that indigenous fungal BCA would stand a better chance at establishment in the rhizosphere and eventually managing target pathogens as compared to exotic/introduced isolates (Monfort et al. 2006).

Soil incorporation with compost is an effective initial indicator of the potential capacity of the soil to support microbial populations and diversity (Rao et al. 2019). Soil organic matter content is essential in the growth and survival of fungi functioning as a primary source of nutrition (Allison 1973). Previous studies (Khan et al. 2011; Rizvi et al. 2018) have shown that incorporation of organic matter (in the form of manure, compost or saw dust) with the application of fungal BCA better manages other fungal-caused diseases RKN while consequently improving plant growth parameters. The objective of this study was to investigate the potential of indigenous nematophagous fungi as BCA against *M. enterolobii* population densities in vitro and in vivo on dry bean under with and without compost incorporation.

## Materials and methods

### Indigenous fungal biotypes

Soil samples were collected from the rhizosphere of various fruit trees and vegetable plots with no previous record of fungal inoculations at North-West University, agricultural research farm, Molelwane, Mmabatho (25° 40.459′ S, 26° 10.563′ E). The soil samples were then transported to the laboratory in sterile polythene zip-lock bags for further tests. Serial dilutions were made from 10 g in 100 mL soil suspension with sub-subsamples being cultured on Petri dishes containing potato dextrose agar (PDA) media (Biolab, Lawrenceville, Georgia, USA) (39 mg L^−1^) treated with Streptomycin (0.5 g L^−1^) (Mast Diagnostics, UK). Plates were then incubated at 25 ± 2 ℃ for 3 days, and afterwards fungal colonies growing on media were transferred and sub-cultured onto new PDA media to obtain pure biotypes (Dada and Aruwa 2014). Seven-day-old pure biotype mycelia of respective fungal isolates were collected from the colony edges and microscopically examined. The hyphae and spore color, size, and shape were used to identify the different species (Gams and Bissett 1998). Isolates with distinct morphological features were further identified at a molecular level using sequencing of internal transcribed spacer (ITS) gene region, with ITS1 and ITS2 sub-regions, with the help of taxonomists at Inqaba Laboratory, Tshwane, Gauteng Province, SA.

### Indigenous nematophagous fungi parasitism on *Meloidogyne enterolobii* eggs and J2

*Meloidogyne enterolobii* raised on a nematode susceptible tomato cv. “Hotstuff” (MayFord Seeds (Pty) Ltd, Pretoria, SA) were extracted for the experiment. When required, dark brown-colored egg masses of *M. enterolobii* were obtained from 2-month-old tomato plants, egg masses from the roots were dislodged using a tooth pick and placed in a Petri dish containing 1% NaOCl solution before being rinsed with distilled water. Eggs displaced in water were counted and used in the egg hatch bioassay.

For the effects of indigenous nematophagous fungi on *M. enterolobii* second-stage juvenile (J2) mortality, the nematode egg masses in distilled water were placed in an incubator set at 25 ± 2℃. Juveniles that hatched in the first 24 h were discarded while those that hatched in the subsequent 48 h were used in the bioassay. The bioassays were conducted by separately exposing *M. enterolobii* eggs and J2 to previously identified pure fungal isolates; *Aspergillus terreus*,* Talaromyces minioluteus*,* T*. *sayulitensis*, *Trichoderma ghanense*, and* T*. *viride*. Before the bioassays, the fungi were growth on PDA for seven-days, with spores obtained by scraping the mycelial growth using a fiber glass hockey stick placed into sterile Petri dishes containing 10 mL of distilled water. The spore suspensions were standardized to 1 × 10^5^ spores/mL for all fungal isolates. Distilled water was used as an untreated control, with each experiment replicated five time. A 100 egg/J2 of *M. enterolobii*/5 mL were separately exposed to the five fungal isolates in Petri dishes. Treatments were arranged in a complete randomized design (CRD) and incubated at 25 ± 2℃. At 24, 48, 72 and 96 h, fungal isolates’ ovicidal and nematicidal activities on *M. enterolobii* were observed. This was achieved by examining and counting eggs/J2 that showed direct mycelial penetration and disintegration relative to untreated control (Singh and Mathur 2010). All experiments were carried out twice for data validation.

### Indigenous nematophagous fungi effects on *Meloidogyne enterolobii* population and growth of dry bean

In the net-house, 25-cm diameter plastic planting pots were filled with 2:1 (v/v) ratio of river sand to field clay soil which was steam pasteurized at 100 ℃ for an hour. A 6 × 2 factorial experiment was laid out in a randomized complete block design (RCBD) with five replications. The first factor consisted of the five fungal species, *A*. *terreus*,* T*. *minioluteus*,* T*. *sayulitensis*,* T*. *ghanense*,* T*. *viride*, and untreated plants, while the second factor consisted of organic and no-organic soil treatments. Organic soil treatments consisted of Compost (Garden Master, SA) which was steam sterilized at 121℃ for an hour and used at a rate of 100 g per pot (translating to approximately 22 680 kg per ha) was incorporated. Following a modified procedure by Coninck et al. (2020), dry bean cv. “PAN 148” seeds were sterilized with 10% NaOCl solution for 5 min, washed three times for 5 min with sterile distilled water. The seeds were put on sterile paper towel and allowed to air dry for 24 h inside a laminar flow cabinet (FILTA-MATIX). Three surface sterilized seeds per pot were sown at 5 cm depth and kept moist until emergence, thereafter there were irrigated with 300 mL of water every second day. At two-leaf stage, two seedlings were thinned out from each pot, leaving only one seedling. At 14 days after seedling emergence, each plant was inoculated with 300 M*. enterolobii* eggs and J2. A day after nematode inoculation, fungal species treatments were applied as a liquid suspension formulated by mixing from Petri dishes of the respective fungal BCA in 2 L of distilled water. Spore concentrations for all fungal species were adjusted to 1 × 10^7^ spores/mL before applying, and 5 mL of suspension was applied per plant at the cardinal hole near the foot of the plant using a plastic syringe. LAN (28) was applied twice, 7 and 10 days after seedling emergence during the growing season at a rate of 0.6 g/plant in 300 mL water per pot.

At 45 days after inoculation, chlorophyll content, plant height, and stem diameter were measured per plant. The plant shoot was cut and weighed for fresh mass before drying them for 72 h in an oven set at 60℃ to obtain the dry mass. The root systems were removed from the soil, immersed in water to remove soil particles, blotted dry, and weighed. Root galling were assessed using a North Carolina Differential Scale (NCDS) of 0 = no galls, 1 = 1 − 2 galls, 2 = 3 − 10 galls, 3 = 11 − 30 galls, 4 = 31 − 100 galls and 5 = 100 galls per root system (Taylor and Sasser 1978). The nematodes were extracted from the entire root system using the maceration and blending process (Marais et al. 2017). The soil in each pot was well mixed, and 250 mL of soil samples were collected, with nematodes extracted from it using a sugar floatation and centrifugation process (Marais et al. 2017). Eggs + J2 from root and soil were counted under a stereomicroscope (Olympus Corporation Tokyo 163–0914, CX23RTFS2). The final population (Pf) density of nematodes was calculated by adding total eggs and J2 from the root system to total J2 from the soil. Reproductive factors [RF = Pf divided by the initial population (Pi) inoculated] indicators of nematode's ability to reproduce were computed (Kayani and Mukhtar 2018). The trials were conducted twice, January − March and October − December 2022, for data validation.

### Statistical analysis

In vitro data for *M. enterolobii* egg hatch inhibition and J2 parasitism were subjected to analysis of variance (ANOVA) through SPSS software (IBM SPSS 2022). Means were separated using the Fisher’s least significant difference (LSD) at P ≤ 0.05 using SPSS software. For the in vivo net house trials, eggs + J2 in root, J2 in soil, gall rating index, RF data were log10(x + 1) transformed. All, in vivo data were subjected to ANOVA. Means were separated using the LSD at *P* ≤ 0.05 using SPSS software (Gomez and Gomez 1984).

## Results

### Identification of fungal biotypes

Macroscopic and microscopic morphological characteristics were used to examine the isolated fungi found in the soils. Based on morphological and molecular characteristics, a total of six indigenous fungal species were isolated belonging to three genera, *Aspergillus*, *Talaromyces* and *Trichoderma. Aspergillus* genus had one species, *A. terreus,* two species come from *Talaromyces, T. minioluteus* and* T. sayulitensis* while *Trichoderma* had two species, *T. ghanense* and *T. viride* (Table 1).

**Table 1:** Molecular identification on indigenous fungal species

GenBank accession number	Nearest BLAST match	Max. identity (%)	E-value
MT316343.1	*A. terreus*	99.83	0.00
MN788118.1	*T. minioluteus*	97.69	0.00
MZ014549.1	*T. sayulitensis*	100.00	0.00
MF078652.1	*T*. *ghanense*	100.00	0.00
MW456070.1	*T*.* viride*	100.00	0.00

### In vitro fungal parasitism of *Meloidogyne enterolobii* eggs and J2

**Fig. 1:**
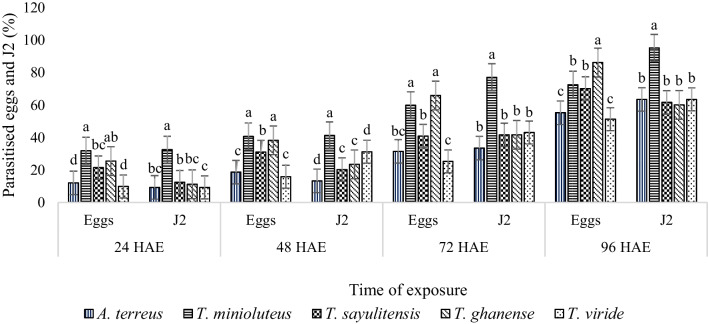
The efficacy of fungal species on *M*. *enterolobii* eggs and J2 in vitro. Small bars represent the standard errors of the means. Means, in the same exposure time and nematode stage, followed by the same letter are not significantly different (*P* ≤ 0.05)

There were significant differences (*P* < 0.01) among the fungal species treatments on both egg and J2 parasitism across all time intervals. Percentage parasitism of both eggs and J2 increased with the increase in exposure time. All fungal species were effective in parasitizing over 50% of inoculated *M. enterolobii* eggs and J2 after 96 HAE. *Talaromyces minioluteus* and *T. ghanense* had the highest percentage egg parasitism, whereas, *A. terreus* and *T. viride* had the least at 24, 48 and 72 HAE. At 96 HAE, *T. ghanense* had the highest percentage egg parasitism (86%), followed by *T. minioluteus* (72%) and *T. sayulitensis* (70%), while *A. terreus* (55%) and *T. viride* (51%) had the least parasitism percentage. *Talaromyces minioluteus* had the highest J2 parasitism at all exposure times, while the other fungal species were second best at 24, 72, and 96 HAE. At 48 HAE, *T. viride* was second best, followed by *T. sayulitensis* and *T. ghanense,* whereas *A. terreus* had the least J2 percentage parasitism (Fig. 1).

### In vivo effects of nematophagous fungi on *Meloidogyne enterolobii* population densities

**Fig. 2:**
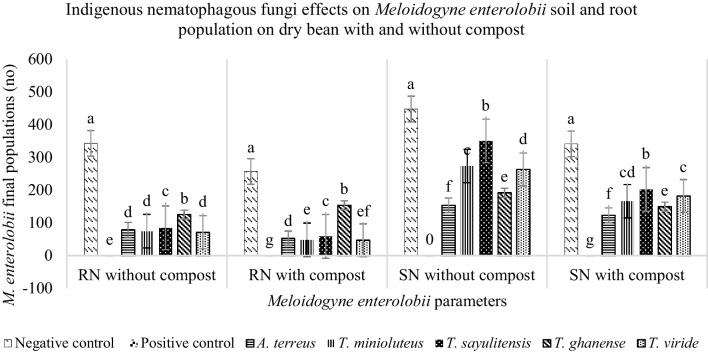
Effects of nematophagous fungi on *Meloidogyne enterolobii* population in soil (SN) and root (RN) with and without compost

There were significant differences (*P* ≤ 0.01) in root and soil *M. enterolobii* Pf when considering both fungal species and compost as individual and combined sources of variation. Dry bean roots had a lower final population of *M. enterolobii* when compared to soil across all fungal species treatments. Dry bean plants treated with *T. ghanense* had the highest final nematode population in roots, irrespective of compost application, followed by *T. sayulitensis,* with the other three, *A. flavus, T. minioluteus and T. viride*, performing the same in soils without compost, whereas, in soils with compost, *A. flavus* had higher nematode population when compared to *T. minioluteus* and *T. viride,* which were not different from each other. In soils with or without compost, *T. sayulitensis* treated soils had the highest final nematode population densities when compared to the other four fungi, followed by treatments with *T. minioluteus*, then *T. viride*, with *A. flavus* having the least when soils have compost amendments. In soils not amended with compost, *T. minioluteus* and *T. viride* had similar effects on Pf, whereas other fungi performance comparisons were similar to those in the absence of compost (Fig. 2).

**Fig. 3:**
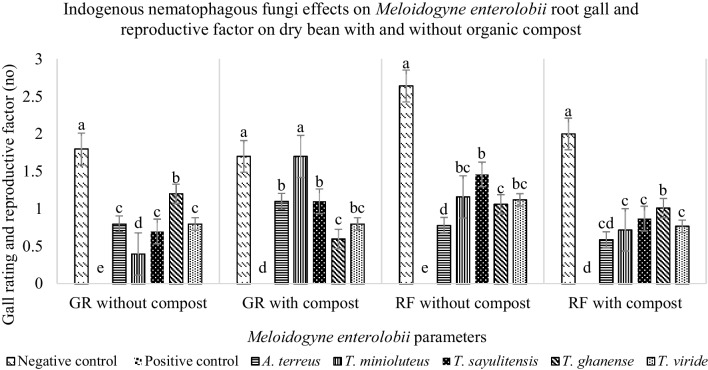
Root gall (GR) rating and *M. enterolobii* reproductive factor (RF) as influenced by indigenous nematophagous fungi with and without compost incorporation

There was significant difference (*P* < 0.05) under the combined factors of fungal species and compost and fungal species as a single factor on dry bean root gall rating; however, the compost as a single factor had no significant (*P* = 0.082) effect on the same variable. There was also no significant difference (*P* = 0.106) between the two independent trials; for that reason, results from one trial are presented herein. Generally, root galling in the presence of compost was higher than in the absence among the *Aspergillus* and *Talaromyces* genera, with *T. minioluteus* having the overall highest galling index. A complete deviation is seen within the *Trichoderma* genus, where the administration of *T. ghanense* resulted in comparatively higher galling without compost than with compost, while incorporation of compost with *T. viride* had no effect as the galling was the same with or without compost (Fig. 3).

Nematode reproductive factor was not aligned to the observed galling, with *M. enterolobii* having a higher RF in the absence of compost among the *Aspergillus* and *Talaromyces* genera than in the presence of the same. The spread of variation on nematode reproduction within plants treated with *Trichoderma* was not wide, while overall, *A. terreus* treated plants had the lowest nematode reproduction in the presence of compost. The positive control recorded the highest nematode reproduction in the absence of compost, high soil and root egg and J2 populations without compost incorporation (Fig. 3).

The only significant differences were observed in plants grown in soils amended with compost for both dry bean height and chlorophyll content (*P* < 0.01 and *P* = 0.026, respectively). Dry bean pod number, pod mass, and dry shoot biomass gave significant differences (*P* < 0.01) under compost as a single source of variation and no significant differences the fungal species nor the combined effect with compost. The negative ( −) control had the overall highest height (81.3 cm), followed by *A. terreus* and *T. minioluteus* (both averaging 76 cm) under compost presence. The shortest dry bean plant height was recorded in the + control treatments, and chlorophyll content was, however, highest under the + control treatment without compost incorporation (108.41 mmol/m^2^), whereas *T. ghanense* followed with almost half chlorophyll content, at 64.7 mmol/m^2^. *Aspergillus terreus* treated plants had the least chlorophyll content at 31.85 mmol/m^2^ (Table 2).

**Table 2:** Pooled mean data for impact of *Meloidogyne enterolobii* on dry bean plant heigh (PH) in cm, chlorophyll content (CC) in mmol/m^2^, stem diameter (SD) in mm, pod number (PN), pod mass (PM) in g, shoot fresh mass (FM) in g and shoot dry shoot mass (DM) in g inoculated with indigenous nematophagous fungi with and without compost (C) under net house at 56 days after inoculation

PH	PH + C	CC	CC + C	SD	SD + C	PN	PN + C	PM	PM + C	FM	FM + C	DM	DM + C
52.58^bc^	63.79^ cd^	108.41^a^	40.77^b^	2.52^ab^	2.49^b^	3.80^bc^	7.90^b^	17.32^bc^	33.03^a^	20.78^b^	16.24^ cd^	3.84^b^	4.73^b^
63.14^a^	81.39^a^	98.74^b^	57.36^a^	3.03^a^	4.10^a^	10.15^a^	12.33^a^	31.66^a^	36.71^a^	28.12^a^	27.03^a^	4.11^a^	5.71^a^
58.30^b^	76.04^b^	55.83^d^	31.85^c^	2.76^ab^	2.13^b^	4.90^bc^	6.90^b^	22.48^b^	28.97^ab^	20.06^b^	17.56^c^	3.76^b^	5.15^a^
47.79^d^	76.04^b^	55.35^d^	39.88^b^	2.50^ab^	2.97^b^	3.90^bc^	7.50^b^	16.64^bc^	33.84^a^	13.57^d^	18.27^c^	3.24^b^	5.07^a^
52.29^bc^	67.30^c^	58.05^ cd^	37.38^b^	2.36^ab^	2.06^b^	4.40^bc^	7.00^b^	20.51^b^	29.40^ab^	28.96^a^	16.05^ cd^	3.37^b^	4.49^ab^
54.22^bc^	69.68^c^	64.70^c^	39.74^b^	2.53^ab^	2.91^b^	5.60^b^	8.00^b^	23.14^b^	33.03^a^	16.89^c^	20.86^b^	3.80^b^	4.77^ab^
46.85^d^	67.27^c^	60.97^c^	37.68^b^	2.50^ac^	2.27^b^	6.10^b^	6.40^b^	23.12^b^	28.63^ab^	17.18^c^	20.57^b^	3.17^b^	5.00^b^

^z^Column means followed by the same letter were not different (*P* ≤ 0.05) according to Fisher’s least significant difference

## Discussion

### Indigenous nematophagous fungi effects on *Meloidogyne enterolobii* in vitro

#### Effects on *Meloidogyne enterolobii* eggs

In vitro and in vivo investigations were carried out to explore the mechanism of indigenous nematophagous fungi suppression capabilities on *M. enterolobii*. Overall, the data showed that the fungal species were effective in suppressing *M. enterolobii* growth and reproduction. Using a stereomicroscope, the relationship between the nematophagous fungi isolates and *M. enterolobii* was examined, and the parasitism mechanism of the five nematophagous fungi was assessed. The surfaces of the infected eggs could clearly be seen surrounded by fungal spores and hyphae from *Talaromyces* and *Trichoderma*, respectively. Eggshells enclosed by spores dissolved/disintegrated, whereas those encased in hyphae mainly retained their form, although the inside were almost entirely destroyed. This suggested that these fungal species produced secondary metabolites to aid spores and hyphae locate and penetrate the eggshells.

Fungal species from these genera, *Aspergillus*, *Talaromyces*, and *Trichoderma* have been extensively explored on *Meloidogyne* parasitism (Abootorabi and Naraghi 2017; Dharshini et al. 2021; Farhat et al. 2022; TariqJaveed et al. 2021; Wang et al. 2022; Ying et al. 2019). But what distinguishes the current analyzed species is that they are suited to the fluctuating climatic conditions of the North-West Province, and possibly other semi-arid warm temperate regions. The current observations suggest that the *Aspergillus* isolate studied could have potential to inhibit egg hatching process directly and indirectly, and subsequently halt *Meloidogyne* reproduction. On the other hand, the finding that most of the unhatched eggs in fungal treatments were surrounded by fungal spores submits that spore penetration was the primary parasitism mechanism.

The varying degrees of egg parasitism effects observed among fungal species’ treatments can be attributed to both direct and indirect modes of actions. Thus, directly through spore/mycelia penetration and indirectly by secreting nematode-toxic metabolites/enzymes. *Talaromyces* is an endophytic genus with species known to produce toxic metabolites such as, siderophore ferrirubin, herquline B, and 3-O-methylfunicone (Vinale et al. 2017). These metabolites could have induced the observed *Meloidogyne* eggshell degradation. Further research on fungal secondary metabolites would be extremely helpful in determining if fungal inhibitors produced into the environment, such as toxic substrates, are the cause of the discrepancy between the rate of egg infection and the decreased rate of egg hatching.

Egg disintegration of those treated with *Trichoderma* was noted throughout the experiment but more prominently on the last day of observation. This is supported by Goswami et al. (2008) and Singh and Mathur (2010) who proved how disintegration was linked to myco-toxic compound release from the fungal species as well as active feeding throughout experimentation. Biological management activity of fungal BCA is due to its physical characteristics such as the adhesive nature of spores and mycelia in addition to the virtual arrangement of nets, knobs, mycelial rings and branches (Askary 2015) that are effective in trapping nematode eggs.

#### Effects on *Meloidogyne enterolobii* J2

According to findings of the current study, *M. enterolobii* J2 exposure to *A. terreus* filtrate decreased J2 mobility and viability rates. Jang et al. (2016) reported that *A. niger* F22 produced potent metabolites, identified as oxalic acid, that have nematicidal effects on *M. incognita* and *M. hapla*. *Aspergillus* species are known to produce a variety of secondary metabolites which act as nematicidal agents either alone or in enhancement of other bio-control species (Siddiqui et al. 2004). Singh and Mathur (2010) also determined in vitro efficacy of indigenous fungi; where *A*. *terreus* was found to be highly toxic against *M*. *incognita* J2, causing high mortality around 68%. Similarly, Göze Özdemir and Arıcı (2022) reported that the culture filtrate of *A*. *niger* at various concentrations showed nematicidal activity against *M. incognita* J2.

With little movement, it seemed as though these nematodes were paralyzed. Most J2 treated with *Talaromyces* became stiff and reduced to a dead husk. Apart from visible adhesion to fungal structures, there were recorded incidences of J2 without close contact to spores nor mycelia which had dead juveniles. *Talaromyces minioluteus* and *T*. *sayulitensis* showed evidence of extracellular enzymes secretion that played an important role in the infection process of eggs as they enabled the respective fungus to degrade *M. enterolobii* eggshells.

Microscope examinations indicated that parasitism and protease generated during the infection process were most likely the key mode of action that functioned as the first phase of attack in vitro against *Meloidogyne* J2. *Trichoderma* conidia attached or parasitized the surface of *M. enterolobii* J2, germinated a high number of hyphae and spores, pierced the integument, and reproduced on J2 surface. Several mechanisms, including antibiosis, competition, mycoparasitism, and enzymatic hydrolysis, have been proposed for *Trichoderma* bio-control potential against plant pathogens (Guzmán-Guzmán et al. 2023; Sood et al. 2020; Zin and Badaluddin 2020).

### Tri-trophic interactions among *Meloidogyne* + fungal species + dry bean

#### Effects of *Meloidogyne enterolobii* on dry bean with fungal species treatments

There were qualitative differences among experimental units which can be attributed to the different BCA treatments. This was observed on harvested pods under different fungal BCA treatments, showing differences in maturity and pod shape. Despite there being no significant difference between dry bean growth and yield parameters, it can be noted that there was a range such as *A. terreus* having the highest mean height (60.96 cm) and stem girth (2.96 cm) (Table 2) while the control had lowest mean number of pods (4.00) and lowest mean pod mass (7.90 g). The two *Trichoderma* species had different effects on dry bean, while *T. ghanense* had the highest mean number of pods, *T. viride* had the lowest proportion of diseased leaves. Thus, it shows the well evidenced ability of the species within the genus to buffer by protecting plants against nematode attack through jasmonic acid (C_12_H_18_O_3_)-regulated defense pathways activation which induces resistance after colonizing plant roots (Martínez‐Medina et al. 2017).

In terms of compost incorporation, *T. sayulitensis* treatment had the highest overall mean fresh biomass (40.49 g) corresponding to a higher mean dry biomass (4.17 g) compared to all other treatments (Table 2), showing its potential to increase productivity while playing a role in bio-protection against *M. enterolobii*. This effect of plant-growth promotion through biomass accumulation by *Talaromyces* species was reported on bamboo (An-qi et al. 2019).

When the fungus develops alongside the plant roots, its hyphae also create a physical barrier, which is a challenging step for nematodes. Producing fungal metabolites and inducing resistance are two additional methods in addition to direct antagonism that *Trichoderma* species use to suppress *Meloidogyne* (Goswami et al. 2008). This could explain why some *M. enterolobii* J2 successfully penetrated dry bean roots, evidenced by galling index, but there were still no detected above-ground nematode infection symptoms. It is further noteworthy, that successful nematode penetration, does not always guarantee successful feeding site establishment. This is explainable by the fact that even nematode resistant host are occasionally penetrated by *Meloidogyne* J2, such have been categorized as hosts exhibiting post-penetration resistance (Desmedt et al. 2020; Ramatsitsi and Dube 2020; Ramatsitsi and Ramachela 2023).

#### Effects of nematophagous fungi on *Meloidogyne enterolobii* on dry bean in vivo

The efficacies of the test fungal species were further assessed on dry bean in the net-house. The development of fewer root galls also resulted in considerable decrease in the *M. enterolobii* Pf. The ability of the fungal species to prevent the formation of feeding sites for *M. enterolobii* was amply proven. This observation is supported by the 51–86% eggs that were parasitized across the five examined fungal species in vitro (Fig. 1). The ability of these fungi to inhibit the establishment and reproduction of the *M. enterolobii* feeding site was as effective as the already commercialized *P. lilacinum* PL251, indicating the potential for use as biological control agents. According to the galling index and Pf, *A. terreus*, *T. minioluteus*, *T*. *sayulitensis*, *T*. *ghanense*, and *T*. *viride* treatments clearly reduced the severity of the condition.

According to the results of the current study, it was clear that using *A. terreus* diminished the attraction of *A. terreus*-treated dry bean roots to *M. enterolobii* and greatly lessened nematode penetration. Some nematodes that were able to penetrate dry bean roots that had been treated with *A. terreus* failed to establish a feeding site. The effect of *Trichoderma* isolates on the reduction in nematode penetration was higher than *Aspergillus* but lower than that of the *Talaromyces*. The decrease in penetration brought about by *A. terreus* supports earlier research demonstrating that bio-control fungi are potential choices for nematode control (Le et al. 2009).

The nematophagous fungi showed significantly higher nematicidal activities with the incorporation of compost. It has been suggested that fungi emit hazardous compounds into the environment in which they thrive (Hyde et al. 2019; Manzar et al. 2022; Thambugala et al. 2020). A similar trend has been established by previous research, when the incorporation of compost increased efficacy of nematophagous fungi against *Meloidogyne* species on different crops (D’Errico et al. 2022; Rizvi et al. 2018). The incorporation of compost appears to have improved the efficacy of fungal bio-control against *M. enterolobii.* The larger N additions paired with barley straw resulted in fungal growth rates that peaked at roughly 12 times the control value, whereas the lower level with no N addition resulted in an eightfold rise. There was a noticeable effect of N addition, with the strongest addition increasing fungal growth by 35% when combined with barley straw and 15% when combined with alfalfa (*Medicago sativa* L.) (Rousk and Bååth 2007).

## Conclusion

Soil drenching with *A. terreus*, *T. minioluteus*, *T*. *sayulitensis*, *T*. *ghanense*, and *T*. *viride* in the net-house exhibited a considerable control impact on *M. enterolobii*. This interaction suggests a novel method for bio-control of RKN in dry bean producing regions. This investigation established the mechanism of *M. enterolobii* egg and J2 parasitism by the test fungal species. Considering that these isolates frequently exhibit more receptivity than exotic ones, it is important to consider how well they may be used in subtropical regions with comparable climatic conditions. In order to achieve a higher nematode control, further research should be conducted to determine the metabolites/enzymes secreted by these species.

## Data Availability

The datasets generated during and/or analyzed during the current study are available within the article.
